# Extracellular Nanovesicle Enhanced Gene Transfection Using Polyethyleneimine in HEK293T Cells and Zebrafish Embryos

**DOI:** 10.3389/fbioe.2020.00448

**Published:** 2020-06-11

**Authors:** Zhenzhen Zhang, Kai Wen, Chao Zhang, Fabrice Laroche, Zhenglong Wang, Qiang Zhou, Zunfeng Liu, Jan Pieter Abrahams, Xiang Zhou

**Affiliations:** ^1^Department of Science, China Pharmaceutical University, Nanjing, China; ^2^Institute of Veterinary Medicine, Jiangsu Academy of Agricultural Sciences, Nanjing, China; ^3^School of Traditional Chinese Pharmacy, China Pharmaceutical University, Nanjing, China; ^4^Centre for Carbohydrate Recognition and Signalling, Department of Molecular Biology and Genetics, Aarhus University, Aarhus, Denmark; ^5^Department of Orthopaedics, Tianjin First Central Hospital, Nankai University, Tianjin, China; ^6^State Key Laboratory of Medicinal Chemical Biology, Key Laboratory of Functional Polymer Materials, College of Pharmacy, Nankai University, Tianjin, China; ^7^C-CINA, Biozentrum, Universität Basel, Basel, Switzerland

**Keywords:** gene transfection, extracellular nanovesicles, zebra fish, polyethyleneimine, efficiency

## Abstract

It is a hot topic to improve efficiency and decrease toxicity of gene transfection reagents. The extracellular nanovesicles (EVs) that are released by cells play an important role in intercellular communication and are naturally designed for genetic exchange between cells. Here, we show that the EVs have a large beneficial effect in polyethyleneimine (PEI)-mediated transfection of a GFP-encoding plasmid into HEK293T cells. An improvement of transfection efficiency of ~500% and a decrease in toxicity were observed in a specific concentration range of PEI. The EVs also greatly improved the transfection of the same plasmid into zebrafish embryos. To verify the generality of this gene transfection approach, we also tested the cell viability and gene transfection efficiency using two other plasmids (EpTEN and ELuc) and in another cell line (A549). The measured increase in transfection efficiency makes EV a promising candidate for enhancement of the quality of current PEI-based transfection technique.

## Introduction

The importance of gene transfection has grown immensely over the past years, as gene therapy may remedy many diseases caused by genetic disorders. Gene transfection *in vivo* or *ex vivo* has been the subject of many recent studies: cancer therapy, neurodegenerative disorders, and blindness and diabetes mellitus (Kent and Krolewski, 2016; SáFilho et al., 2017; Cideciyan et al., 2018; Yuan et al., 2019). Transfection efficiency and toxicity are the key factors of therapeutic effectiveness. Cells can communicate by releasing extracellular nanovesicles (EVs) in extracellular space, which play important role in cell–cell communications (Johansson et al., 2018).

Gene transfection vectors can help genes to overcome cellular barriers, which include synthetic and viral vectors (Saffari et al., 2016). Viral vectors and their clinical trials in human gene therapy have saved human lives (Poletti et al., 2018). Viral vectors show high transfection efficiency, while they exhibit low gene-carrying capacity and limited cell-targeting specify (Hernandez-Garcia et al., 2014). Moreover, the public health implications of each viral vector remain to be estimated on a case-by-case basis (Alessia et al., 2013).

Compared to the viral vectors, the synthetic ones are mostly positively charged polymers, which can have different cell type specificities than viruses. They can bind DNA to form positively charged complexes with sizes between 40 and 150 nm, which do not show risks of genetic damage and are therefore safe to use (Hernandez-Garcia et al., 2014). For example, polyethyleneimine (PEI) is a well-characterized polycationic gene transfection vector toward nucleic acids (DNA, RNA, miRNA, or siRNA) (Kent and Krolewski, 2016). In this paper, we investigated the effect of extracellular nanovesicles (EVs) for enhancing the gene transfection of PEI in mammalian cells and zebrafish embryos. However, synthetic cationic polymers have shown to be cytotoxic *in vitro* (Kadlecova et al., 2012) and *in vivo* (Storka et al., 2013) at elevated concentrations, due to cell damage from a cationic charge density of polycations (Kadlecova et al., 2012).

There are several cellular barriers for gene transfection. The first cellular barrier for gene transfection is cellular uptake, which can be overcome by using a positively charged gene carrier/DNA complex (Mosquera et al., 2018). The complex inside the cell will be trapped into the endosome/lysosome. The DNA/carrier complexes that have managed to escape this vesicular trafficking pathway are then faced with the challenge of the complex structure of cytosol. The filamentous structures in the cytosol make it difficult for DNA/carrier complexes to diffuse freely through the cytosol (Hernandez-Garcia et al., 2014; Saffari et al., 2016). Dissociation of DNA and its carrier may be necessary to make it possible to reach the nucleus, while there is a risk for DNA to be degraded by the nucleases (Hernandez-Garcia et al., 2014). Transporting to the cell nucleus is another cellular barrier, because it is difficult for plasmid DNA to enter the nucleus when the cell is not in a mitotic state (Alton et al., 2014; Remaut et al., 2014; Maity and Stepensky, 2017).

Gene transfection efficiency has been improved by the development of various approaches based on overcoming different barriers. Gene delivery can be made more specific by using cell surface receptor-specific ligands, like peptides (Hao et al., 2019), antibodies (Saqafi and Rahbarizadeh, 2019), and vitamins (Song et al., 2015). For an endosomal escape, the use of stearylated INF7 modified liposomes (Dolor et al., 2018) or cholesterol-containing lipoplexes have been shown as a superior design for delivery systems (Hattori et al., 2015). There are many ways to improve the transport of DNA through the cytosol. Synthetic fusion proteins can be used to link molecular motor proteins to the DNA/carrier complexes or DNA. In this, way the cargo can be transported to the nucleus so that cytosolic trafficking of the DNA can be improved (Garcia-Gradilla et al., 2013). Another way for transporting plasmid DNA across the nuclear envelope is to coat the plasmid DNA with nuclear localization sequences (Remaut et al., 2014; Maity and Stepensky, 2017). Moreover, plasmid DNA can be targeted to the nuclear compartments of specific cell types by including special DNA nuclear targeting sequences in the constructs. Although progress has been made for the rational design of synthetic gene delivery vectors, their transfection efficiency is lower than that of the viral vectors (Hernandez-Garcia et al., 2014).

Intercellular communication can occur via release of membrane vesicles into the extracellular space (Johansson et al., 2018), and all living systems contain these EVs with unique structural, mechanical, and biochemical characteristics (Betzer et al., 2018; Dolor et al., 2018). The EVs are either nanovesicles that are originated from the endosomal compartment of cells (Johansson et al., 2018), or the shedding vesicles directly budding from the plasma membrane (Cocucci and Meldolesi, 2015). One possible way of intercellular communication is assumed to occur via release of EVs from the cell membrane of one cell and fusing with another cell. This behavior of EV could be used to overcome the barriers in gene transfection, such as entering cell membrane, endosomal escape, and perhaps even nuclear uptake. Recent studies indicates that EVs can serve as good candidates for drug delivery and gene transfection (Shandilya et al., 2017; Betzer et al., 2018). In most of these studies, the drug or genes are always encapsulated into EVs and delivered in cells (Betzer et al., 2018). However, there has been no investigation of using EVs to improve the transfection efficiency of a synthetic gene transfection reagent, such as PEI. In this paper, the EVs in HEK293T cell culture medium were isolated by differential centrifugation method, and the effect in gene transfection using PEI as transfection vector in Hek293T cells, A549 cells, and in the zebrafish embryos were investigated.

## Results

### Extracellular Vesicle Preparation and Characterization

**Figure 1:**
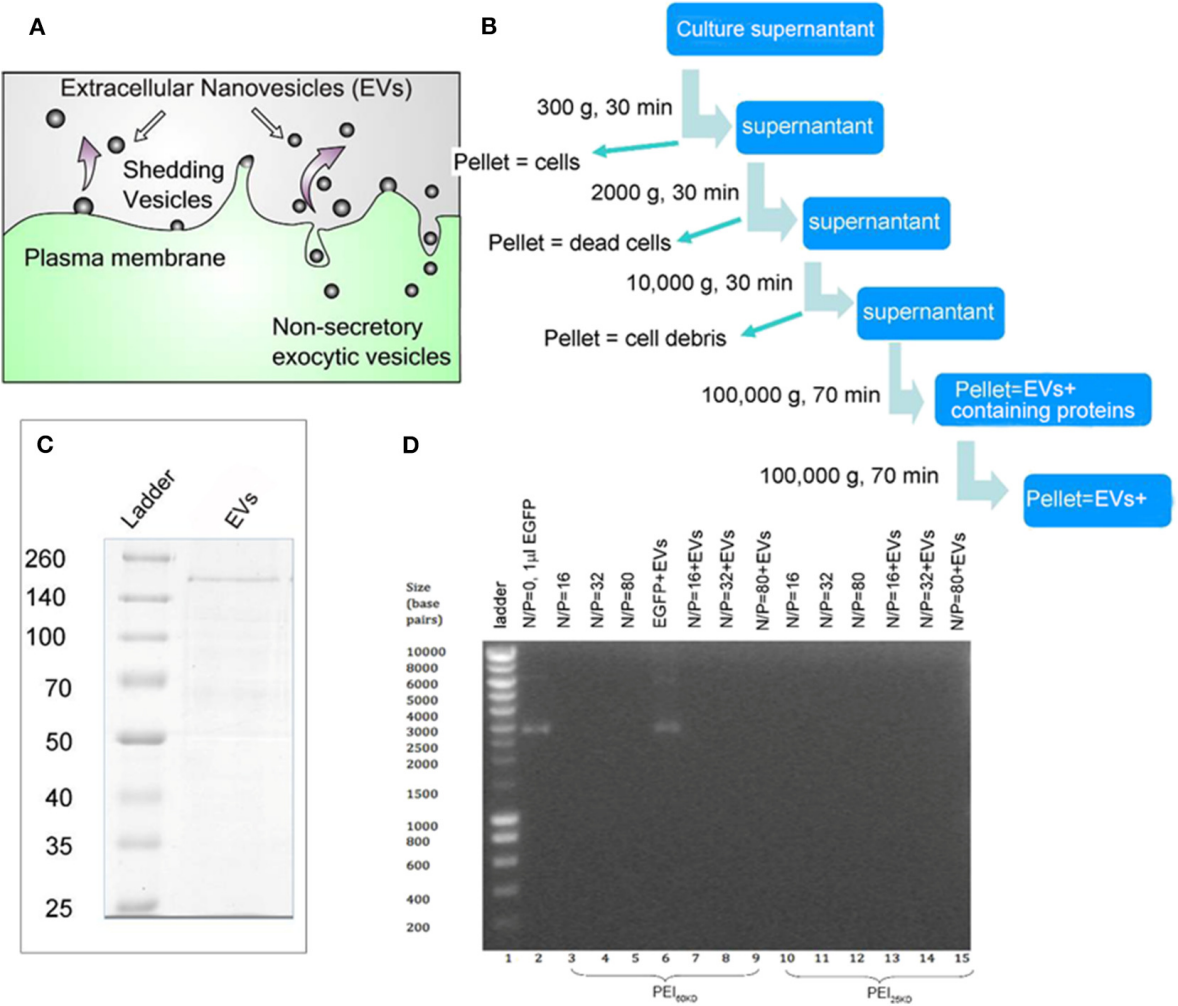
**(A)** Schematic representation for EV generation from cells. **(B)** EV isolation process by multiple centrifugation. **(C)** SDS PAGE gel of EVs, showing a thick band with molecular weight of ~200 kD and a few thin bands between 50–200 kD using Coomassie Blue staining. **(D)** Band-shift assay of DNA, PEI/DNA complexes, and PEI/DNA/EVs complexes, at different N/P ratios (the molar ratio of nitrogen in PEI to phosphor in DNA). In **(D)** PEI_60kD_ was used in lanes 3–9 and PEI_25kD_ was used in lanes 10–15.

EVs were isolated by serial centrifugation, as schematically represented in Figures 1A,B. This process was performed multiple times so that enough EVs were collected for gene transfection. EVs isolated from the same batch of culture supernatant of HEK293T cells were used for enhancement of gene transfection of PEI in HEK293T cells and in zebrafish embryos. The PEIs with a molecular weight of 25 and 60 kD were used, which were abbreviated as PEI_25kD_ and PEI_60kD_, respectively.

**Figure 2:**
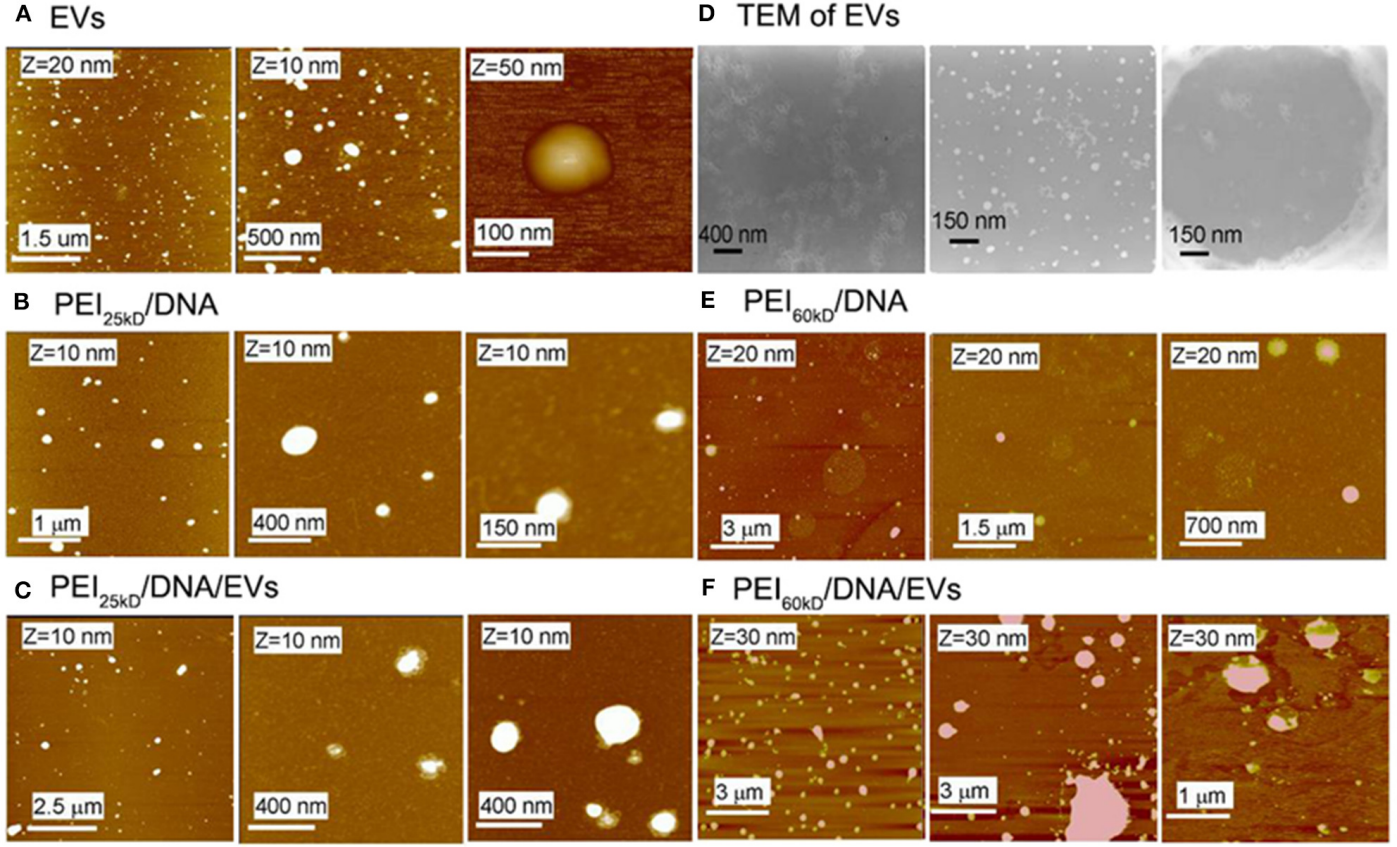
AFM images of **(A)** EVs, **(B)** PEI_25kD_/DNA, **(C)** PEI_25kD_/DNA/EVs, **(E)** PEI_60kD_/DNA, **(F)** PEI_60kD_/DNA/EVs, and **(D)** TEM images of EVs. In **(A)**, the EVs were adsorbed on positively charged mica that was functionalized with (3-amino- propyl)triethoxysilane. In **(B,C,E,F)**, the complexes were adsorbed on freshly cleaved mica. In **(D)**, the left image shows EVs that were fixed with PFA, stained with PTA, and adsorbed on positively charged carbon grid; the middle image showed the EVs that were fixed with PFA, stained with PbAc, and adsorbed on positively charged carbon grid; the right image shows the EVs that were fixed with PFA, stained with PTA, and adsorbed on formvar grid.

The protein content in the EVs was analyzed to be 3 g/mL^−1^ by Bradford assay (Supporting Information S1, S2). The SDS PAGE of the EVs shows a thick band of molecular weight of ~200 kD and some light bands between 50 and 200 kD (Figure 1C). The EVs were characterized using transmission electron microscopy (TEM) after fixation with paraformaldehyde (PFA) and stained using phosphotungstic acid (PTA) or lead acetate (PbAc), as shown in Figure 2D. The EVs negatively stained with PTA on positively charged carbon grids or formvar grids (Figure 2D, left and right images) showed typical cup-like spherical vesicle structures, while EVs positively stained with PbAc showed round particles with size ranging from 20 to 100 nm. EVs, PEI/DNA complexes, and PEI/DNA/EVs complexes were analyzed by band-shift assay and atomic force microscopy (AFM). The band-shift results show that two DNA bands are visible in lanes two and six, corresponding to molecular weight of ~3,000 and 7,000 kD (Figure 1D). In other lanes, no DNA was detected, indicating the full capture of DNA in the PEI/DNA and the PEI/DNA/EV complexes.

**Figure 3:**
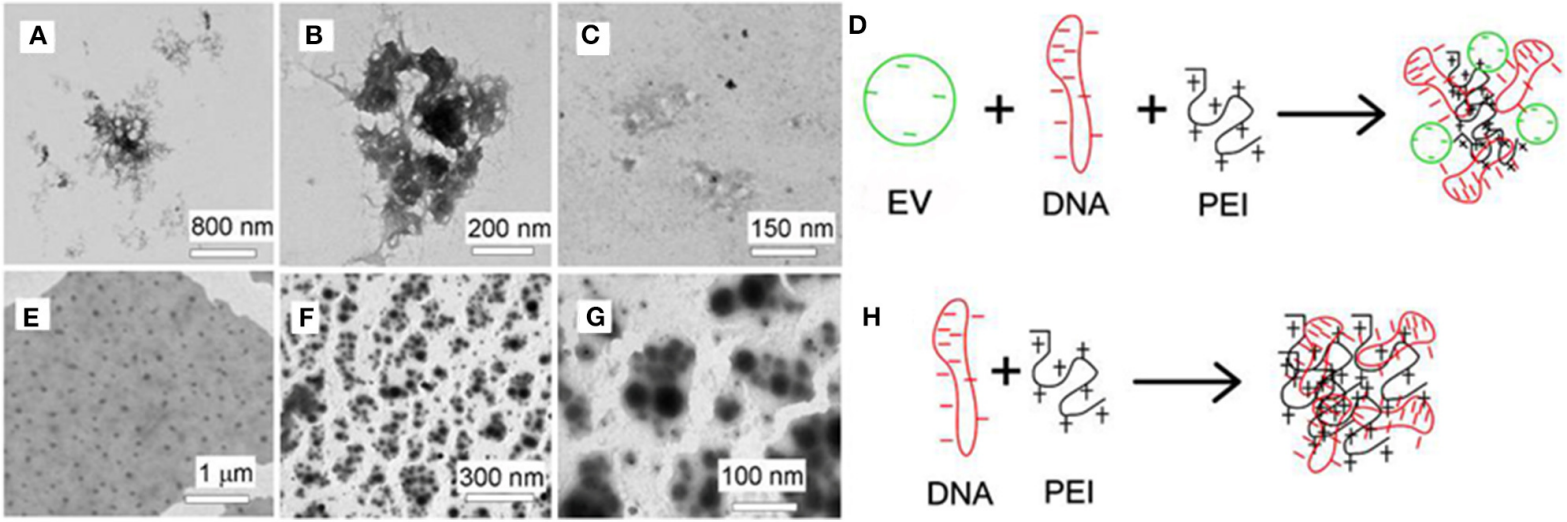
**(A–C)** TEM images of PEI_25kD_/DNA/EVs; **(E–G)** TEM images of PEI_25kD_/DNA; **(D,H)** Schematic representation of the complex formation of **(D)** EVs/DNA/PEI and **(H)** PEI/DNA.

The AFM images in Figure 2A show that the EVs have size of 50–150 nm. The formation of the PEI/DNA and the PEI/DNA/EVs complexes can also be confirmed by AFM (Figures 2B,C,E,F). PEI_25kD_/DNA complexes were detected as round particles with size ranging from 50 to 300 nm (Figure 2B, left). The magnified images show that a few DNA strands appear at the margins of the particles (Figure 2B, middle and right). PEI_25kD_/DNA/EVs complexes were also detected as round particles with a size of 50–200 nm (Figure 2C). The magnified images in Figure 2C, middle and right show that the DNA strands stuck out of the complexes. We further investigated the structure of PEI_25kD_/DNA and PEI_25kD_/DNA/EVs complexes using TEM, which were stained with PTA (Figure 3). It can be seen that the EVs embedded in the PEI_25kD_/DNA/EVs complex, which were connected by thread-like molecules (Figures 3A–D). In contrast, the PEI_25kD_/DNA EVs complex showed compact structure (Figures 3E–H). It can be seen that both PEI/DNA and PEI/DNA/EVs complexes formed particles, with DNA observed on the surface of the complexes, indicating that DNA can form complexes with PEI in the presence of EVs. The zeta potential of PEI25_kD_/DNA/EVs was from +20 to +36 mV for N/P ratio from 2 to 32, indicating a positive charge of the polyplexes (Figure S3).

### Extracellular Vesicle Enhanced Gene Transfection of PEI in HEK293T Cells

**Figure 4:**
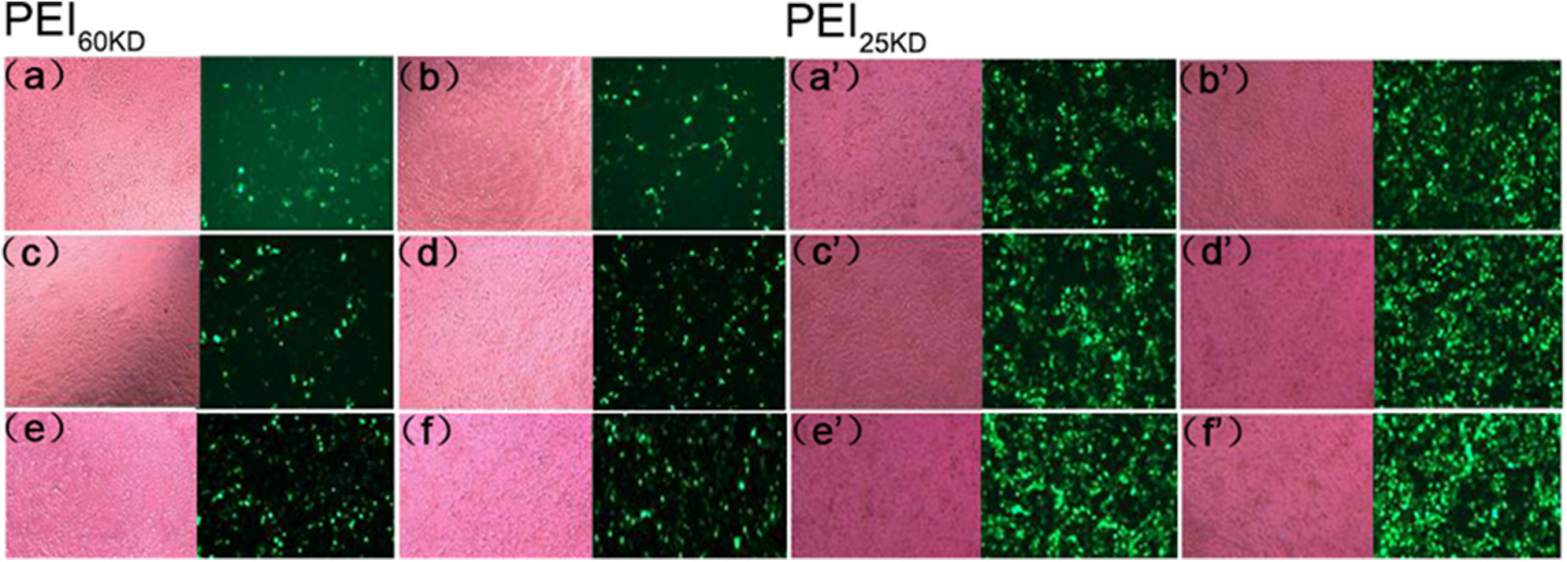
Optical (left) and fluorescent (right) images of HEK293T cells transfected with plasmid EGFP using **(a–f)** PEI_60kD_ at N/P = 80 and **(a'–f')** PEI_25kD_ at N/P = 160, at a post transfection time of 48 h. EV solution of different volume (0, 0.5, 1, 2, 5, and 10 μL) were added into each well of the 96-well plate for images **(a–f)** and for **(a'–f')**, respectively. The protein concentration in EV solution was 3 ng/μL^−1^ All the images were taken at the same exposure time (500 ms).

Gene transfection experiments were performed for plasmid EGFP using PEI_60kD_ and PEI_25kD_ at various N/P ratios and different amount of EVs in HEK293T cells. Transfection efficiency and cell toxicity were qualitatively assessed by using light microscopy and fluorescence microscopy. To show the effect of EVs on PEI gene transfection, light and fluorescence images of transfection of plasmid EGFP in HEK293T cells with PEI_60kD_ at N/P ratio of 80 and PEI_25kD_ at N/P ratio of 160 with different amount of EVs are shown in Figure 4.

Figure 4 indicates that a substantial number of GFP positive cells present for both PEI_60kD_ and PEI_25kD_ transfected cells without adding EVs. In the optical images, we did not see obvious floating dead cells, indicating little toxicity. Upon including EVs, the number of GFP positive cells increased noticeably (Figures 4a,b,a',b'). With increasing the amount of EVs, the number of GFP positive cells increased gradually (Figure 4), indicating a clearly positive effect of EVs on PEI gene transfection. In the optical images, we did not observe an obvious increase in the number of dead cells.

**Figure 5:**
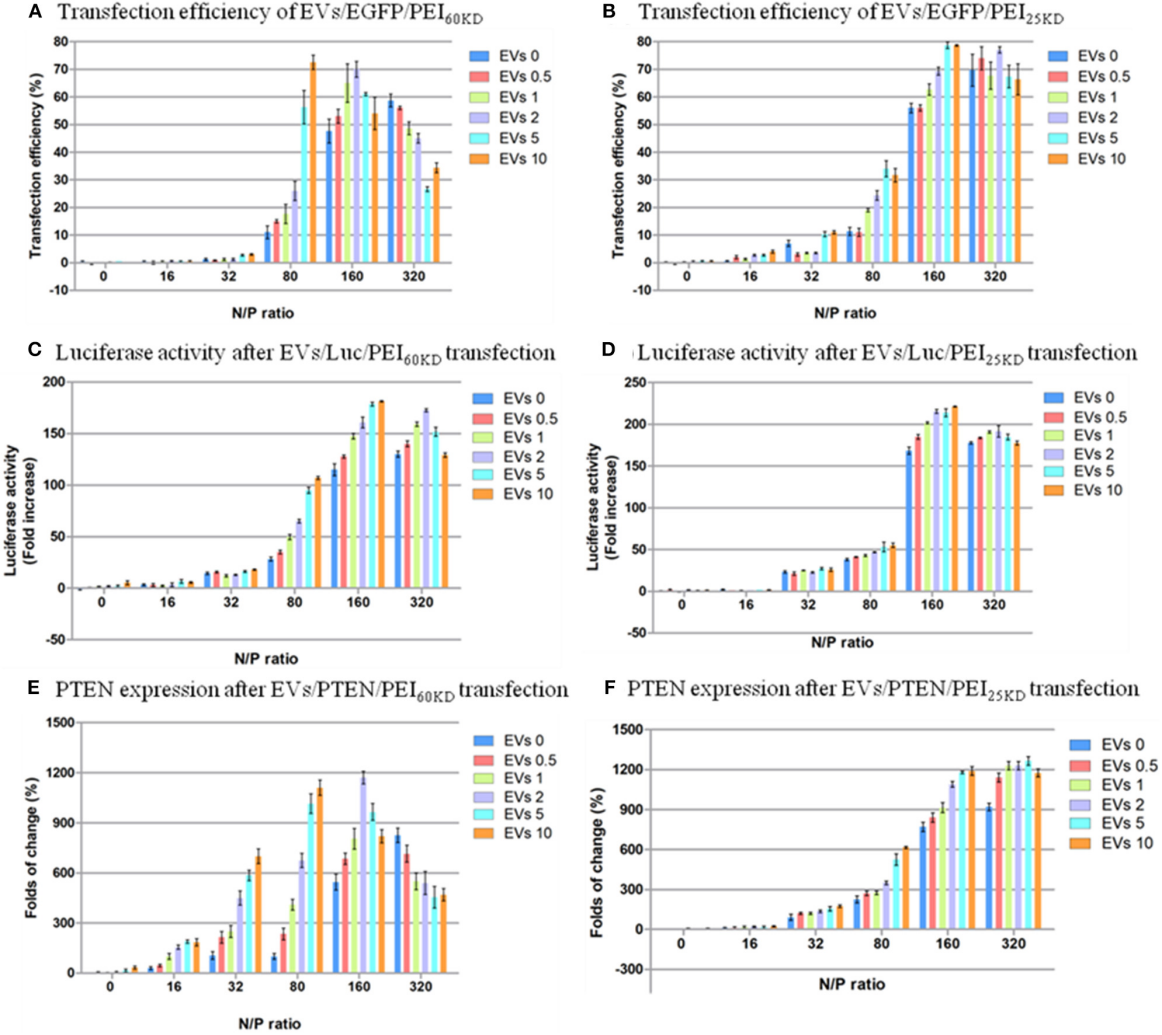
Transfection efficiency of HEK293T cells by plasmid EGFP using **(A)** PEI_60kD_ and **(B)** PEI_25kD_; Luciferase activity after transfection using **(C)** PEI_60kD_ and **(D)** PEI_25kD_; PTEN expression after transfection using **(E)** PEI_60kD_ and **(F)** PEI_25kD_; determined by MTT assay, at a post transfection time of 48 h for different N/P ratios. EV solution of different volume (0, 0.5, 1, 2, 5, and 10 μL) with protein concentration of 3 ng/μL^−1^ were added into each well of the 96-well plate.

**Figure 6:**
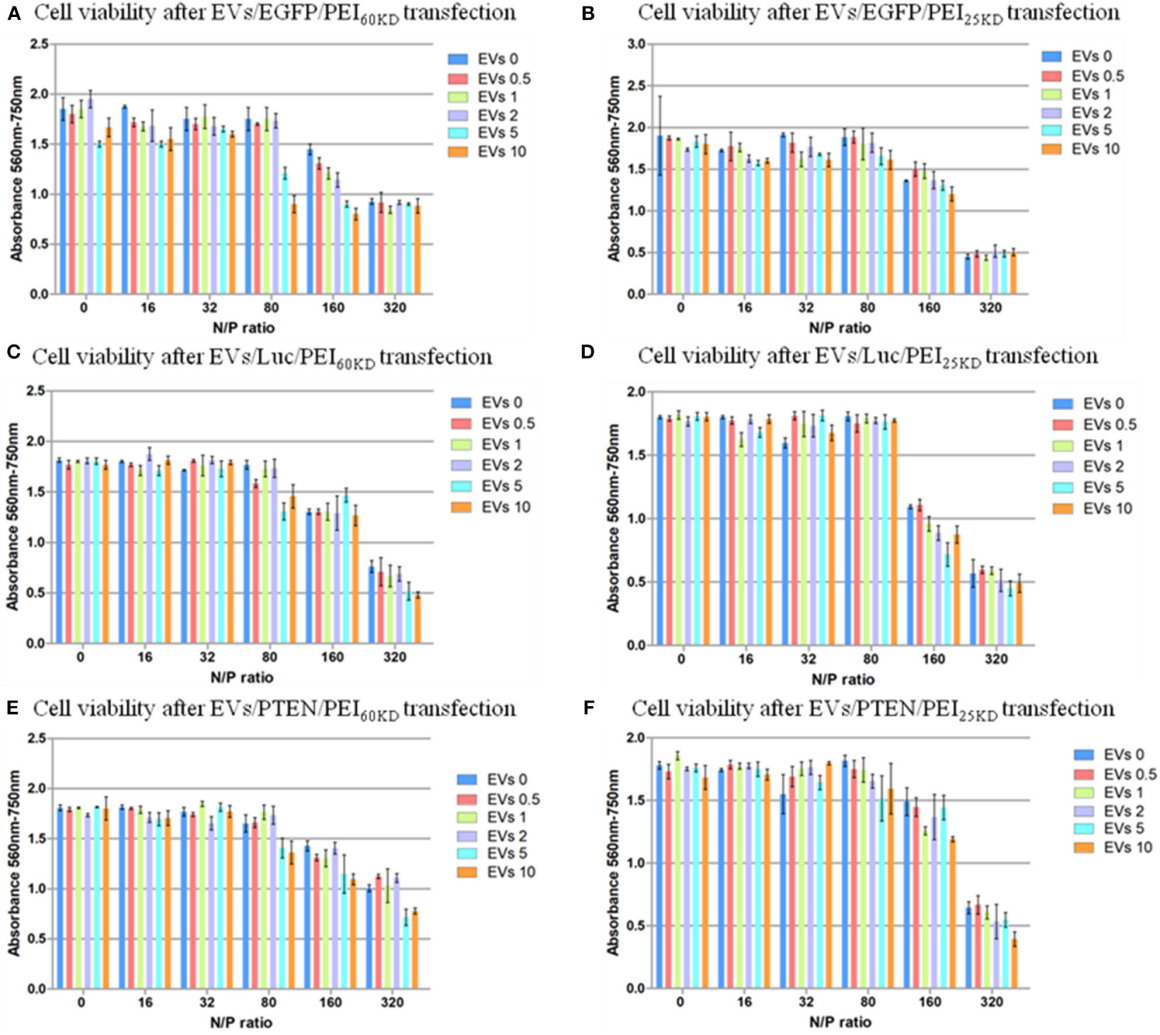
Cell viability of HEK293T cells transfected with plasmid EGFP using **(A)** PEI_60kD_ and **(B)** PEI_25kD_; Cell viability of HEK293T cells transfected with plasmid ELuc using **(C)** PEI_60kD_ and **(D)** PEI_25kD_; Cell viability of HEK293T cells transfected with plasmid EpTEN using **(E)** PEI_60kD_ and **(F)** PEI_25kD_, determined by MTT assay, at a post transfection time of 48 h for different N/P ratios. EV solution of different volume (0, 0.5, 1, 2, 5, and 10 μL) was added into each well of the 96-well plate. The protein concentration in EV solution was 3 ng/μL^−1^.

However, these pictures only provide anecdotal evidence. Quantitative transfection efficiency measurements were performed by fluorescence-activated cell sorting (FACS), and quantitative cell viability measurements were performed by MTT assay. The results of the FACS measurements of transfection with PEI_60kD_ and PEI_25kD_ are given in Figure 5, and the cell viability results are shown in Figure 6. The transfection efficiency remained zero when only DNA was used. If EVs were added, there was no change in the transfection efficiency (Figure 5). At N/P ratios under 32 the transfection efficiency was very low (<11%) for PEI_60kD_ and PEI_25kD_.

The transfection efficiency using PEI_60kD_ was lower than that using PEI_25kD_ when no EVs were added (Figure 5). In both cases there was a clear increase in transfection efficiency as the N/P ratio increased from 0 to 320, in the absence of EVs. Furthermore, the effect of adding EVs on the transfection efficiency of PEI depended on the N/P ratio. For N/P ratio from 16 to 160, an obvious positive trend in the transfection efficiency could be observed with increasing the amount of EVs (Figure 5A). The positive effect of EVs on transfection efficiency of PEI_60kD_ and PEI_25kD_ was observed at N/P ratio of 80: The transfection efficiency of PEI_60kD_ increased from 12 to 73% (508% increase) by adding 10 μL of EVs, and the transfection efficiency of PEI_25kD_ increased from 11 to 35% (218% increase) by adding 5 μL of EVs. For N/P ratio of 320, the transfection efficiency decreased as EVs were added for transfection using PEI_60kD_.

The toxicity of PEI at N/P ratios of 160 and 320 are visible (Figure 6). Clusters of cells were observed at these high N/P ratios, which was a clear sign of unhealthy cells under stress (data not shown). The toxicity of adding EVs into the transfection of PEI showed dependence on N/P ratio. No significant difference in toxicity could not be detected by adding EVs (0.5–10 μL) into transfection of PEI_25kD_ and PEI_60kD_ for N/P ratios from 0 to 32. For the transfection of PEI_25kD_ and PEI_60kD_ with N/P ratios of 80 and 160, the toxicity did not increase by adding <2 μL of EVs, and it decreased by adding 5 and 10 μL of EVs. For the transfection of PEI_25kD_ and PEI_60kD_ with N/P ratio of 32, adding EVs showed negligible effect on toxicity, where the cell viability was low.

### Extracellular Vesicle Enhanced Gene Transfection of PEI in Zebra Fish Embryos

**Figure 7:**
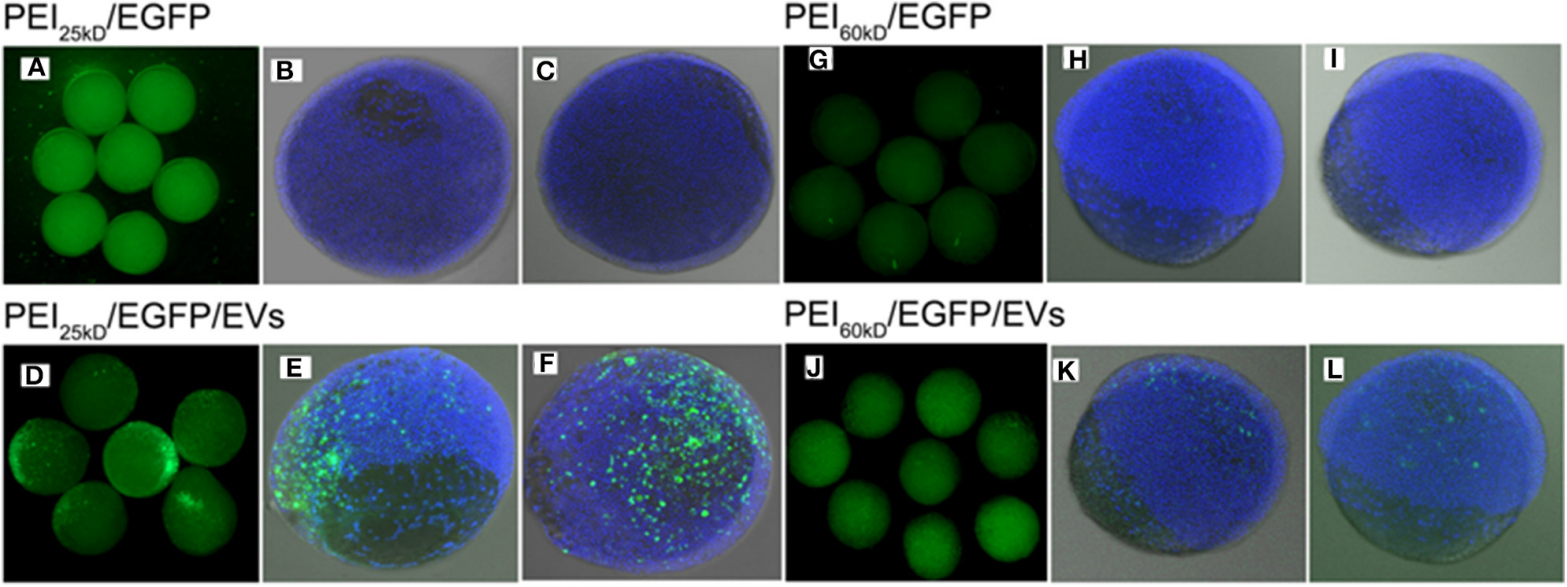
Fluorescence images of **(A–C)** PEI_25kD_/EGFP, **(D–F)** PEI_25kD_/EGFP/EVs, **(G–I)** PEI_60kD_/EGFP, and **(J–L)** PEI_60kD_/EGFP/EVs in zebrafish embryos 8 h after injection.

Zebra fish are an excellent model organism in molecular biology due to the low cost maintenance, short generation time, and translucency of embryos for *in vivo* imaging (Gerlai et al., 2017). Therefore, we tested the influence of EVs on transfection of zebra fish embryos with PEI_60kD_ and PEI_25kD_ using plasmid EGFP as reporter gene. Using the knowledge we gained from the HEK293T transfection experiments described above, the feed ratio of N/P ratio of 160, 2 μL of EV solution, and 0.2 g of DNA was used for transfections of PEI_60kD_ and PEI_25kD_. Micro-injection inside the embryo interlayer in the first cell stage led to EGFP over expression 8-h after injection, an overview image of zebrafish embryos and two confocal fluorescence images of PFA fixed embryos are shown in Figure 7. Transgene delivery efficiency can be estimated with Hoechst 33342 fluorescent signal.

From overview and confocal images in Figure 7, it can be seen that the transfection using either PEI_25kD_/DNA or PEI_60kD_/DNA did not lead to high-level expression of EGFP in zebrafish embryos. In comparison, a substantial number of EGFP positive cells can be seen for the case of PEI_25kD_/DNA/EVs, as shown in Figures 7D–F. For PEI_60kD_/DNA/EVs, a few positively GFP transfected cells can be seen, indicating that the EVs can improve the gene transfection of PEI in zebra fish (Figures 7J–L). From Figures 7D–F,J–L, it can be seen that gene transfection using PEI_25kD_/DNA/EVs was more efficient than using PEI_60kD_/DNA/EVs.

By counting ~200 embryos, we observed ~90% EGFP-positive transfection (~90% embryos with some EGFP positive cells), with viability of ~80% for the case of PEI_25kD_/DNA/EVs. This efficiency is much higher than that of PEI_25kD_/DNA (<10% in our experiments). The PEI_60kD_/DNA/EVs showed a transfection efficiency of 60%, which is much higher than that of PEI_60kD_/DNA injection (<10%). This indicated that the PEI/DNA/EVs system is a very promising gene transfection system for zebra fish embryos.

## Discussion

### Extracellular Vesicle Characterizations

The bands in the SDS page in Figure 1D are very faint, indicating that there is only non-significant amount of protein present in the EV sample. The results correlate with the data obtained by the Bradford assay, which indicated a protein concentration of 3 μg/mL^−1^. Clearly, the serial ultracentrifugation process eliminated most of the contaminating proteins from the EV sample. There is not enough data available to determine the identity of the remaining proteins.

It is a great challenge to distinguish shedding vesicles from EVs, as the EV populations recovered from extracellular fluids are inevitably mixed. The characteristics of the EVs and the ratio of shedding vesicles vs. EVs can change under different preparation conditions. Many physicochemical and composition properties of EVs and shedding vesicles, like size, and density, are very similar. Therefore, they remain closely associated in the subcellular fractions isolated by differential centrifugation (Cocucci and Meldolesi, 2015).

The EVs can stick to positively charged mica, indicating a negatively charged surface of EVs. They can be seen in the AFM images in Figure 2A as particles with size of ~50–150 nm. The TEM images of EVs suggested sizes of 20–100 nm. This size distribution is similar to the size of EVs reported in the literature (Cocucci and Meldolesi, 2015). The negatively stained EVs showed a “cup-like” shape, which is a typical structure of hollow-sphere micelles in TEM (Johansson et al., 2018).

The EVs in AFM appear somewhat larger than those in TEM; this is probably due to the tip convolution effect during AFM scanning. The AFM cantilever has a diameter of 10–20 nm; therefore, the particle size is always broadened by twice the tip diameter. The band-shift assay of the EVs mixed with EGFP plasmid shows that the bands of EGFP have almost the same intensity as that in pure EGFP, indicating negligible DNA binding capacity of the EVs by simple mixing. Two bands were observed in lanes two and six, which are attributed to the different conformations of EGFP due to supercoiling.

PEI is a positively charged gene delivery vector with high DNA-binding activity (Hernandez-Garcia et al., 2014). The band-shift assay and AFM images confirmed the formation of condensed PEI/DNA complexes (Figures 1D, 2, 3). In the band-shift assay, no band is visible at a positive N/P ratio. This is because the positively charged DNA/PEI complex will not travel through the gel from negative to positive in electrophoresis. The existence of PEI/DNA complexes was also confirmed by AFM (Figures 2B,E) (Hernandez-Garcia et al., 2014). PEI_60kD_ formed a more condensed structure with DNA compared to the PEI_25kD_. There are DNA strands sticking out of the PEI_25kD_/DNA complex, and no DNA strands sticking out for the PEI_60kD_/DNA complex.

Since EVs are negatively charged, it would be reasonable to expect that PEI has less DNA-binding capacity when EVs are added to the sample. For PEI/DNA and PEI/DNA/EVs with investigated N/P ratios, no noticeable difference in DNA binding capacity was observed in band-shift assay. Clearly, all DNA molecules still bound to the PEI with addition of EVs. This might be different at a rather low N/P ratio where limited amount of PEI would be used, in which case the EVs could compete with DNA binding by PEI.

The AFM images demonstrate that the PEI/DNA/EVs complexes were less condensed than the PEI/DNA complexes (Figures 2B,C). This indicates that EVs participated in condensation of DNA on PEI. Moreover, the PEI/DNA/EV complexes in the AFM images were larger than the PEI/DNA complex (Figure 3). This also indicates complex formation of DNA and EVs on PEI. The PEI/DNA/EVs complexes were less condensed than the PEI/DNA complexes, with more DNA exposed to the solvent, which may facilitate gene transfection. In Figure 3, the PEI/DNA/EV complex also show a rather loose structure compared to the PEI/DNA complex. The particles are surrounded by the thread-like molecules, which would be ascribed to the EVs complexed with DNA and PEI. From the above results of band-shift assay, AFM and TEM, a schematic representation demonstrates the possible formation mechanism of PEI/DNA and PEI/DNA/EVs complexes (Figures 3D,H).

The surface charge of the polyplexes is rather important to the gene delivery process. The positively charged nature of the polyplex will facilitate the association to the negatively charged cell membrane. Then we measured the surface charge of the PEI/DNA/EVs polyplexes at a different N/P ratio and different EV volume, as shown in Figure S3. The polyplexes formed by PEI_25kD_ and PEI_60kD_ share similar trends. The zeta potential is below −10 mV at N/P ratio of 0 (no PEI added). This is reasonable that both DNA and EV are negatively charged. By increasing the EV amount, we observed a further decrease of zeta potential value from −10 to −14 mV. The zeta potential of PEI25_kD_/DNA/EVs increased from +20 to +36 mV, with an increase of N/P ratio from 2 to 32, indicating a positive charge of the formed polyplexes. The zeta potential tended to decrease with increasing EV at low N/P ratio values (e.g., N/P = 2 and 4). While the zeta potential did not change too much for EV amount at N/P ratios from eight to 32, indicating a strong positively charged nature of the polyplexes.

### Transfection Experiments

Transfection requires more than just adding naked DNA. EVs on its own does not induce transfection, indicating that the positively charged PEI is essential. This enables PEI an effective reagent for internalization of DNA into cells (Mosquera et al., 2018). Moreover, EVs show poor DNA binding activity, as is confirmed by the agarose gel results in Figure 1D.

According to the reports, PEI_25kD_ shows higher transfection efficiency and lower cytotoxicity than PEI_60kD_ (Jiang et al., 2019). This difference in transfection efficiency was confirmed by our results that the transfection with PEI_25kD_ yielded a higher efficiency than for PEI_60kD_ at the same N/P ratio (in the absence of EVs). However, the difference in cytotoxicity between PEI_25kD_ and PEI_60kD_ could not be confirmed from Figure 6, as there was no measurable difference between the cell viability after transfection with PEI_25kD_ or PEI_60kD_ in HEK293T cells (in the absence of EVs). For the above experiments, the difference in molecular weight between the two compounds did not differ enough to cause a measurable difference in the cytotoxicity in HEK293T cells. The molecular weight of PEI used in Sadeghpour et al. (2018) differed more than that in this study.

N/P ratios under 32 yielded a low transfection efficiency (<11%). At these low N/P ratios, however, all the DNA molecules were condensed on the PEI/DNA complex as confirmed by the band-shift assay in Figure 1D. This suggests that DNA binding and condensation is not the only factor that affects gene transfection, but that the amount of PEI available, after binding DNA, is also of influence. As a consistency, we observed increased transfection efficiency for increased N/P ratio. Besides the DNA binding and cell internalization, PEI also plays a very important role in endosomal escape due to its “proton sponge effect” (Sadeghpour et al., 2018). At increasing N/P ratio the cell viability decreased, indicating the toxicity of PEI at high N/P ratios.

Figure 6 shows that the cell viability is almost not influenced by increasing the EV amount when the positively charged polymer are not used, indicating EVs did not show toxicity when mixed with DNA. EVs can help increasing the transfection efficiency of PEI, but also increase the toxicity of PEI transfection at N/P ratio higher than 80. However, the effect of EVs is not strictly linear. There appear to be two major toxic effects—the PEI on its own and the intracellular delivery of DNA. This follows from comparison of Figures 5, 6: (i) increased transfection is accompanied by increased toxicity, (ii) when transfection has reached a plateau, increasing the PEI concentration results in additional cell death. The data suggest that some of the PEI can be substituted by EVs, which reduces PEI dependent, but transfection independent cell death.

According to the results, EVs generally have a positive effect on the transfection efficiency, with the exception at an N/P ratio of 320. The maximum improvement of the efficiency is higher in the transfection for PEI_60kD_ (508%) than for PEI_25kD_ (218%). The positive effect of EVs on the transfection efficiency could be brought on by the less condensed structure of PEI/DNA/EVs complex in comparison to the more compact PEI/DNA complex, which would render DNA more accessible (Figures 2, 3).

To verify the generality of this gene transfection approach, we also tested the cell viability and gene transfection efficiency using two other plasmids (EpTEN and ELuc) and in another cell line (A549). Similar results were obtained for transfecting plasmids of EGFP, EpTEN, and ELuc in cell lines of H293T and A549 (Figure 6, Figure S5). This indicates that our approach is widely applicable for different gene plasmids and for different cell lines.

The zebrafish can be used as a very good animal model for developmental biology studies. However, it is notoriously difficult to be transfected. Zebrafish transgenesis remains difficult because embryo pro-nuclei are not visible and cannot be micro-injected. Therefore, micro-injection at the interlayer between the first cell and the yolk is a method of interest (Zhou et al., 2012). In Figure 7 we show that transfection using either PEI_25kD_ or PEI_60kD_ (in N/P ratios optimized for the HEK293T cells) in the absence of EVs was ineffective. However, when EVs were added, the transfection was much more efficient. This indicates that the EVs really plays a very important role in the transfection system of PEI/DNA/EVs. There are several important differences between developing zebrafish embryos and cell growing in culture, which may explain the inability of PEI on its own to transfect DNA, and the positive role of EVs might play in this process. In zebrafish, the cells develop much faster compared to the HEK293T cells. For example, the zebrafish will develop from one-cell stage to four-cell stage within 1 h, and to 64-cell stage in 2 h (Kimmel et al., 1995). However, the HEK293T cells in culture dish develop much slower, which double every 24 h. Second, the three-dimensional matrix in the inter-cellular environment for the living organism of zebrafish embryos is totally different from the cultured cells. Furthermore, the volume of fertilized egg cell is much large than dish-cultured HEK293T cells. It was reported that EVs are known to play very important role in the inter-cellular communication, there is a possibility that inter-cellular communication through EVs plays an important role during the organism development. Such a positive effect of EVs may motivate their use in PEI-based transfection in zebrafish embryos.

## Conclusions

EVs were successfully isolated from cell culture, which showed size of 50–150 nm by TEM and AFM. The protein content in EVs solution is not significant. The band-shift assay showed that PEI, DNA, and EVs form complexes by mixing, which were less condensed compared to PEI/DNA. Addition of EVs in gene transfection by PEI in Hek293T cells improved the transfection efficiency. The use of EVs in gene transfection of PEI in zebrafish embryos through interlayer injection also improved transfection. The cell viability of adding EVs showed dependence of N/P ratio, with low toxicity at low N/P ratios and high toxicity at high N/P ratios. The detailed mechanism by which EVs assists in gene transfection of PEI needs further studies. Because polycations also occur naturally (Chen et al., 2019), it can be assumed that gene transfection with EVs and natural polycations can also take place in natural systems. Although currently the expected frequency of gene transfection with natural polycations is quite low because of low concentration of natural polycations in natural systems, the use of EVs in such systems may provide a promising all-natural transfection system.

## Experimental

### Preparation of EVs

EVs were isolated from the supernatant of HEK293T cell culture, which have been cultured according to ATCC recommendations in Dulbecco's Modified Eagle Medium (DMEM) supplemented with Penicillin/Streptomycin mixture (pen/strep) and fetal calf serum (FCS). The Penicillin/Streptomycin mixture contains penicillin (5,000 units/mL^−1^) and streptomycin (5,000 g/mL^−1^). The HEK293T cells were seeded at a confluence of 20% and cultured in CO_2_ (5%) and humidity (95%) at 37°C for 72 h before collecting the supernatant. EVs were isolated from the supernatant by serial centrifugation, according to the protocol given in Supporting Information S1, which is an adapted form of the protocol given in the literature (Thery et al., 2001).

### SDS-PAGE, Bradford Assay, AFM, TEM, and Zeta Potential

The protein content of the isolated EV samples was determined by SDS-PAGE and Bradford assay. The Bradford assay was performed on NanoDrop 2000 UV/Vis spectrophotometer (Thermo Scientific). The protocol for the NanoDrop 2000/2000c was described in Supporting Information S2. For SDS-PAGE test, the EV sample (20 μL) was mixed with protein loading buffer (5 μL, 5×) and incubated for 15 min at 95°C, and then the mixture (20 μL) was loaded on the acrylamide gel (9%). The gel ran for 45 min at 200 V and was washed in water for 5 min three times. Then it was stained for 1 h using Coomassie Brilliant Blue and washed twice in water again for 1 h. The gel images were analyzed using the software “Quantity One.”

AFM images were obtained on a multimode AFM (Digital Instruments) at tapping mode at a frequency of 300 kHz. EVs can be captured on a positively charged mica. The positively-charged mica was prepared by incubating a freshly cleaved mica in a freshly-prepared mixture of (3-aminopropyl) triethoxysilane (APTES, 30 L) and H_2_O (30 mL) for 2.5 min, followed by washing with ethanol and H_2_O for three times, and dried with N_2_ flow. The PEI/DNA complex was attached on mica surface by loading the mixture of EGFP (0.04 g), PEI (1.6 L, 0.01%), and PBS (18 L, phosphate buffered saline) on freshly cleaved mica for 5 min, followed by washing with deionized water, and dried by N_2_ flow. Similarly, the PEI/DNA/EVs complex was attached on mica by loading the mixture of EGFP (0.04 g), PEI (1.6 μL, 0.01%), EVs (10 μL), and PBS (8 μL) on freshly cleaved mica for 5 min, followed by washing with deionized water, and dried by N_2_ flow.

TEM images were obtained on a JEOL electron microscope (JEM-1010) at an acceleration voltage of 80 KV. For sample preparation, EVs (10 μL) were mixed with PFA (10 μL, 4%) for 10 min, and incubated on a formvar grid (200 mesh) or a glow discharged carbon grid (400 mesh) for 1 min. Then it was washed consecutively by PTA (1%) or PbAc (1%) and blotted with filter paper.

Zeta potential was measured with a zeta potential analyzer (Zeta Plus, Zetasizer Nano Z system, Malvern Instruments Ltd, UK) at 25°C. One microgram of pEGFP and different amounts of PEI and EV were mixed in 100 μL of PBS, incubated for 20 min, and diluted to 1.5 mL with MillQ water prior to the measurements. The sampling time was set to automatic mode during measurements.

### Gene Transfection in Cells and Zebra Fish Embryos

EGFP-N1 plasmids were isolated from the bacteria of *E. coli* strain DH5α, which were incubated in LB-medium using kanamycine as antibiotic. The pGL3-GAPDH-Luc and pcDNA3.1(+)-PTEN plasmids were isolated from the bacteria of *E. coli* strain DH5α, which were incubated in LB-medium using ampicillin as antibiotic. Identical conditions and the same batch of cells and reagents were used for all the transfection experiments, as was the case for measuring transfection efficiency and cell toxicity of PEI/DNA/EVs. The transfection experiments were performed in HEK293T and A549 cells using EGFP N1, pGL3-GAPDH-Luc, and the pcDNA3.1(+)-PTEN plasmid.

For gene transfection, cells were seeded in 96-well plates (6 × 10^4^ well^−1^) in medium (200 μL). The cells were incubated in 37°C, 5% CO_2_, and 95% humidity for 4 h. The transfection mixture was prepared by mixing PEI, EV, and plasmid (0.2 μg). Cell culture medium (10% FCS, pen/strep) was added until a total volume of 25 μL was reached. The mixture was incubated for ~20 min at room temperature before being added to each well of the 96-well plate. The cells were incubated in 37°C, 5% CO_2_, and 95% humidity for 48 h.

For gene transfection in zebrafish embryos, the zebrafish embryos were micro-injected at the interlayer between the cell and the yolk at one cell stage. Zebrafish were handled in compliance with the local animal welfare regulations and maintained according to the standard protocols (http://ZFIN.org). Single pairs of albino zebrafish embryos were crossed, and single lays were divided into three sets of at least 100 embryos. For each set of embryos ~0.5 nL of the transfection mixture was injected with a Femtojet. The number of embryos injected was in the range of 100–200 for each sample. The experiments were repeated twice. For each embryo, 0.5 nL of the samples were injected with a Femtojet microinjector (Eppendorf) and a micromanipulator using pulled microcapillary needles. For PEI_25kD_ and PEI_60kD_ injections with or without EVs, the following conditions from cellular transfection was used, including EGFP (1.0 μg), corresponding amount of PEI of N/P ratio of 160, EVs (10 μL), and H_2_O (to reach a total volume of 50 μL). The solution was mixed by pipette up and down a few times and incubated for 30 min at room temperature before injection. Zebrafish embryos were allowed to develop in the dark at 28°C in egg water (60 mg/mL^−1^ Instant Ocean sea salts). GFP-positive embryos were screened with a Leica MZ16FA stereo fluorescent microscope and confocal Zeiss LSM5 Exciter/AxioImager. The embryos were fixed with PFA (4%) in PBS-tween (0.1%, PBST) overnight. Counter-staining was performed with Hoechst 33342 diluted at 1 μg/mL^−1^ in PBST overnight. Embryos were dechorionated by hands and immobilized in low-melting agarose (1.5%) for imaging purpose.

### Measurements of PEI/DNA/EVs Transfection Efficiency and Toxicity

The PEI/EGFP/EVs transfection efficiency was determined by FACS. The cells were washed with PBS and tripsinized with 60 μL of trypsin solution prepared by mixing trypsin (25%, 5 mL), PBS (20 mL), and PBS/EDTA (15 mL). After being incubated for 3 min in 5% CO_2_, 95% humidity, and 37°C, cell culture medium (240 μL, 10% FCS, pen/strep) was added to the cells to deactivate the trypsin. After trypsinization, the cells were analyzed by FACS. The lower significance level of the protocol was determined according to the transfection efficiency at an N/P ratio of 0, at which the efficiency is equal to zero and used for each measurement. Supporting Information S3 shows graphs obtained by FACS, with one graph at an N/P ratio of zero (used to determine the significance level) and the other at N/P ratio <0 is given (in which the significance level is obtained). Supporting Information S3 also shows the transfection efficiency given in the FACS protocol. Outliers in the transfection efficiency were indicated by Grubbs test and were removed from the dataset. The significance level used in the Grubbs test is α = 0.05.

Luciferase activity of the cells transfected with PEI/Luc/EVs was determined by luciferase assay (Promega, Bio-Glo™ Luciferase Assay). The protocol of Promega, Bio-Glo™ Luciferase Assay was used.

PTEN and GAPDH (internal control) mRNAs of the cells transfected with PEI/PTEN/EVs were detected by qPCR assay. PTEN Forward Primer: 5′-TGGATTCGACTTAGACTTGACCT-3′; Reverse Primer: 5′-GGTGGGTTATGGTCTTCAAAAGG-3′; GAPDH Forward Primer:5′-GGAGCGAGATC -CCTCCAAAAT-3′, Reverse Primer: 5′-GGCTGTTGTCATACTTCTCATGG-3′.

Cell viability (toxicity) was determined by MTT assay (Promega, Celltiter 96® Non-Radioactive Proliferation Assay). The protocol of Promega, Celltiter 96® Non-Radioactive Proliferation Assay was used, as shown in Supporting Information S4.

## Data Availability Statement

The raw data supporting the conclusions of this article will be made available by the authors, without undue reservation, to any qualified researcher.

## Ethics Statement

Zebrafish were used according to the local animal welfare regulations (http://ZFIN.org).

## Author Contributions

XZ, QZ, and JA were responsible for the experimental concept and design. ZZ, CZ, FL, KW, and ZW carried out the most experiments, characterization, and data analyses. ZL, ZZ, and QZ were responsible for project administration, conceptualization, supervision, formal analysis, funding acquisition, validation, writing original draft, and review and editing. All authors wrote the paper and provided comments and agreed with the final form of the manuscript.

## Conflict of Interest

The authors declare that the research was conducted in the absence of any commercial or financial relationships that could be construed as a potential conflict of interest.

## References

[B1] Alessia C. Arianna M. Fulvio M. (2013). Mechanisms of retroviral integration and mutagenesis. Gene Ther. 24:119. 10.1089/hum.2012.203 23330935

[B2] Alton E. W. Boyd A. C. Cheng S. H. Davies J. C. Davies L. A. Dayan A.. (2014). Toxicology study assessing efficacy and safety of repeated administration of lipid/DNA complexes to mouse lung. Gene Therapy 21, 89–95. 10.1038/gt.2013.61 24196086

[B3] Betzer O. Perets N. Angel A. Motiei M. Sadan T. Offen D.. (2018). Reply to “comment on” *in vivo* neuroimaging of exosomes using gold nanoparticles”'. ACS Nano. 12, 11719–11720. 10.1021/acsnano.8b07946 30995714

[B4] Chen J. Wang K. Wu J. Tian H. Chen X. (2019). Polycations for gene delivery: dilemmas and solutions. Bioconjug. Chem. 30, 338–349. 10.1021/acs.bioconjchem.8b00688 30383373

[B5] Cideciyan A. V. Sudharsan R. Dufour V. L. Massengill M. T. Iwabe S. Swider M.. (2018). Volatile element evolution of chondrules through time. Proc. Natl. Acad. Sci. U. S. A. 115, 8547–8552. 10.1073/pnas.1805055115 30082398 PMC6112700

[B6] Cocucci E. Meldolesi J. (2015). Ectosomes and exosomes: shedding the confusion between extracellular vesicles. Trends Cell Biol. 25, 364–372. 10.1016/j.tcb.2015.01.004 25683921

[B7] Dolor A. Kierstead P. Dai Z. Szoka F. C. (2018). Sterol-modified Peg lipids: alteration of the bilayer anchoring moiety has an unexpected effect on liposome circulation. Chem. Commun. 54, 11949–11952. 10.1039/C8CC05011B PMC6764448 30288531

[B8] Garcia-Gradilla V. Orozco J. Sattayasamitsathit S. Soto F. Kuralay F. Pourazary A.. (2013). Functionalized ultrasound-propelled magnetically guided nanomotors: toward practical biomedical applications. ACS Nano. 7, 9232–9240. 10.1021/nn403851v 23971861

[B9] Gerlai R. Poshusta T. L. Rampersad M. Fernandes Y. Greenwood T. M. Cousin M. A.. (2017). Forward genetic screening using behavioral tests in zebrafish: a proof of concept analysis of mutants. Behav. Genet. 47, 125–139. 10.1007/s10519-016-9818-y 27704300

[B10] Hao F. Dong S. Yang C. Li Z. Cheng Z. Zhong L.. (2019). Targeting and efficient delivery of siRNA using tunable polymeric hybrid micelles for tumor therapy. Anticancer Res. 39, 1169–1178. 10.21873/anticanres.13226 30842146

[B11] Hattori Y. Hara E. Shingu Y. Minamiguchi D. Nakamura A. Arai S.. (2015). siRNA delivery into tumor cells by cationic cholesterol derivative-based nanoparticles and liposomes. Biol. Pharm. Bull. 38, 30–38. 10.1248/bpb.b14-00526 25744455

[B12] Hernandez-Garcia A. Kraft D. J. Janssen A. F. Bomans P. H. Sommerdijk N. A. Thies-Weesie D. M.. (2014). Design and self-assembly of simple coat proteins for artificial viruses. Nat. Nanotechnol. 9:698. 10.1038/nnano.2014.169 25150720

[B13] Jiang C. Qi Z. Jia H. Huang Y. Wang Y. Zhang W.. (2019). ATP-responsive low-molecular-weight polyethylenimine-based supramolecular assembly via host-guest interaction for gene delivery. Biomacromolecules 20, 478–489. 10.1021/acs.biomac.8b01395 30516950

[B14] Johansson H. J. Vallhov H. Holm T. Gehrmann U. Andersson A. Johansson C.. (2018). Extracellular nanovesicles released from the commensal yeast malassezia sympodialis are enriched in allergens and interact with cells in human skin. Sci. Rep. 8:9182. 10.1038/s41598-018-27451-9 29907748 PMC6004016

[B15] Kadlecova Z. Baldi L. Hacker D. Wurm F. M. Klok H. A. (2012). Comparative study on the *in vitro* cytotoxicity of linear, dendritic, and hyperbranched polylysine analogues. Biomacromolecules 13:3127. 10.1021/bm300930j 22931162

[B16] Kent L. N. Krolewski J. J. (2016). Opportunities and challenges in combination gene cancer therapy. Adv. Drug Deliv. Rev. 98, 35–40. 10.1016/j.addr.2015.12.005 26724249 PMC4957561

[B17] Kimmel C. B. Ballard W. W. Kimmel S. R. Ullmann B. Schilling T. F. (1995). Stages of embryonic development of the zebrafish. Dev. Dynam. 203, 253–310. 10.1002/aja.1002030302 8589427

[B18] Maity A. R. Stepensky D. (2017). Nuclear and perinuclear targeting efficiency of quantum dots depends on density of peptidic targeting residues on their surface. J. Control. Release 257, 32–39. 10.1016/j.jconrel.2016.12.031 28042083

[B19] Mosquera J. García I. Liz-Marzán L. M. (2018). Cellular uptake of nanoparticles versus small molecules: a matter of size. Acc. Chem. Res. 51, 2305–2313. 10.1021/acs.accounts.8b00292 30156826

[B20] Poletti V. Charrier S. Corre G. Gjata B. Vignaud A. Zhang F. (2018). Preclinical development of a lentiviral vector for gene therapy of x-linked severe combined immunodeficiency. Mol. Ther. Methods Clin. Dev. 15:257 10.1016/j.omtm.2018.03.002 PMC5918176 29707600

[B21] Remaut K. Oorschot V. Braeckmans K. Klumperman J. De Smedt S. C. (2014). Lysosomal capturing of cytoplasmic injected nanoparticles by autophagy: an additional barrier to non viral gene delivery. J. Control. Release 195, 29–36. 10.1016/j.jconrel.2014.08.002 25125327

[B22] Sadeghpour H. Khalvati B. Entezar-Almahdi E. Savadi N. Hossaini Alhashemi S. Raoufi M.. (2018). Double domain polyethylenimine-based nanoparticles for integrin receptor mediated delivery of plasmid DNA. Sci. Rep. 8:6842. 10.1038/s41598-018-25277-z 29717202 PMC5931586

[B23] Saffari M. Moghimi H. R. Dass C. R. (2016). Barriers to liposomal gene delivery: from application site to the target. Iran. J. Pharm. Res. 15, 3–17. 28228799 PMC5242347

[B24] SáFilho M. F. Gonella-Diaza A. M. Sponchiado M. Mendanha M. F. Pugliesi G. Ramos R. D. S.. (2017). Impact of hormonal modulation at proestrus on ovarian responses and uterine gene expression of suckled anestrous beef cows. J. Anim. Sci. Biotechnol. 8:79. 10.1186/s40104-017-0211-3 29118976 PMC5664832

[B25] Saqafi B. Rahbarizadeh F. (2019). Polyethyleimine-polyethylene glycol copolymer targeted by anti-HER2 nanobody for specific delivery of transcriptionally targeted tBid containing construct. Artif Cells Nanomed Biotechnol. 47, 501–511. 10.1080/21691401.2018.1549063 30810413

[B26] Shandilya S. Rani P. Onteru S. K. Singh D. (2017). Small interfering RNA in milk exosomes is resistant to digestion and crosses the intestinal barrier *in vitro*. J. Agric. Food Chem. 65, 9506–9513. 10.1021/acs.jafc.7b03123 28967249

[B27] Song H. Q. Li R. Q. Duan S. Yu B. Zhao H. Chen D. F.. (2015). Ligand-functionalized degradable polyplexes formed by cationic poly(aspartic acid)-grafted chitosan-cyclodextrin conjugates. Nanoscale 7, 5803–5814. 10.1039/C4NR07515C 25758351

[B28] Storka A. Vcelar B. Klickovic U. Gouya G. Weisshaar S. Aschauer S.. (2013). Effect of liposomal curcumim on red blood cells *in vitro*. Anticancer Res. 33:3629. 24023289

[B29] Thery C. Amigorena S. Raposo G. A. Clayton A. (2001). Current Protocols in Cell Biology. Paris: John Wiley & Sons, Inc. 10.1002/0471143030.cb0322s30 18228490

[B30] Yuan Q. Yi L. Ahmed F. N. Johnathan T. Rui L. (2019). Characteristics and advantages of adeno-associated virus vector-mediated gene therapy for neurodegenerative diseases. Neural Regen. Res. 14, 931–938. 10.4103/1673-5374.250570 30761996 PMC6404499

[B31] Zhou X. Laroche F. Lamers G. Torraca V. Voskamp P. Lu T.. (2012). Glycyrrhetinic acid functionalized graphene oxide for mitochondria targeting and cancer treatment *in vivo*. Nano Res. 5:703. 10.1007/s12274-012-0254-x 29205852

