# Supplementary material for: Extracellular Nanovesicle Enhanced Gene Transfection Using Polyethyleneimine in HEK293T Cells and Zebrafish Embryos
Source: Front Bioeng Biotechnol. 2020 Jun 11;8:448. doi: 10.3389/fbioe.2020.00448 (PMC7300290; doi:10.3389/fbioe.2020.00448)
Supplement: Supplementary file 1 [file Data_Sheet_1.docx]

Supplementary information for

**Extracellular Nanovesicle Enhanced Gene Transfection using Polyethyleneimine**

**in HEK293T Cells and Zebrafish embryos**

**Zhenzhen Zhang,^1,2^ Kai Wen,^1^ Chao Zhang,^3^ Fabrice Laroche,^4^ Zhenglong Wang,^1^ Qiang Zhou,*^5^ Zunfeng Liu, ^6^ Jan Pieter Abrahams,^7^ Xiang Zhou*^1,6^**

^1^ Dr. X. Zhou, Dr. Z. Zhang, K. Wen, Z. Wang
Department of Science, China Pharmaceutical University, 639 Longmian Road, Nanjing, Jiangsu Province, China

^2^ Dr. Z. Zhang

Institute of Veterinary Medicine, Jiangsu Academy of Agricultural Sciences, Nanjing, Jiangsu Province, China

^3^ Dr. C. Zhang

School of traditional chinese pharmacy, China Pharmaceutical University, 639 Longmian Road, Nanjing, Jiangsu Province, China

^4^ Dr. F. Laroche

Centre for Carbohydrate Recognition and Signalling, Department of Molecular Biology and Genetics, Aarhus University, Gustav Wieds vej 10, 8000 Aarhus C, Denmark.

^5^ Dr. Q. Zhou

Department of Orthopaedics, Tianjin first central hospital, Nankai University, Tianjin, 30071 China

^6^ Dr. X. Zhou, Prof. Z. Liu

State Key Laboratory of Medicinal Chemical Biology, Key Laboratory of Functional Polymer Materials, College of Pharmacy, Nankai University, Tianjin, 30071 China

^7^ Prof. J. P. Abrahams
C-CINA, Biozentrum, Universität Basel, CH-4058 Basel, Switzerland

**This PDF file includes the following:**

Supplementary Notes 1 to 5

Supplementary Figure S1-S5

**Supplementary Notes**

**1****. Protocol for** EVs **isolation**

1. Put 25 mL of the culture supernatant in 50 mL centrifuge tubes.
2. Centrifuge, 300 X g, 30 min
   - Take 17.5 mL supernatant, use for step 3
3. Centrifuge, 2000 X g, 30 min
   - Take 12 mL supernatant, use for step 4
4. Centrifuge, 10.000 X g, 30 min
   - Take 8.5 mL supernatant, use for step 5
5. Put the 8.5 mL supernatant from two tubes in one aluminum ultracentrifuge tube, so the total volume per ultracentrifuge tube is 17 mL.
6. Ultracentrifuge, 100,000 g, 70 min
   - Take pellet, resuspend in 20 mL PBS, use for step 7
7. Ultracentrifuge, 100,000 g, 70 min
   - Take pellet, resuspend in 20 μL
8. Add the suspension from every tube in one eppendorf tube and mix by pipetting up and down.
9. Divide the suspension in eppendorf tubes, 100 μL per eppendorf tube.

10. Freeze the EVs suspensions in −80°C, until use.

**2. Protocol for Bradford protein assay using Thermo scientific NanoDrop 2000/2000c**

1. Prepare standard BSA solutions in the following concentrations: 0, 15, 35, 55, and 75 μg mL^-1^

2. Dilute the Bradford reagent by 5 times and put 10 μL of the diluted reagent in six eppendorf tubes (0.5 mL), or six mini-centrifuge tubes (0.2 mL).

3. Add 10 μL of each standard BSA solution and 10 μL of the unknown sample to the reagent and mix by pipetting up and down.

4. Launch the NanoDrop 2000 software and open the Protein Bradford module.

5. Clean the pedestal with a clean laboratory wipe and ethanol, and initialize the instrument by loading 2 μL H_2_O to the pedestal.

6. One by one add 2 μL of the samples to the pedestal and measure. Use the standard concentration of 0 μg mL^-1^ for the blank measurement. Use fresh 2 μL aliquots for each replicate. Measure at least two replicates per standard solution.

**3. Equation used to determine the N/P ratio**


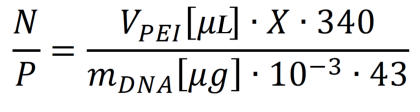


*V*_PEI_ [μL]: Volume of PEI solution in μL

*X*: weight fraction of PEI in PEI solution

*m*_DNA_ [μg]: mass of DNA added in μg

**4. MTT assay protocol**

1. Take out 100 μL of the supernatant from each well of the 96-well culture plate, so that the remaining volume in each well is 100 μL.

2. Add 15 μL of dye solution to each well and incubate in 5% CO_2_, 37°C for 1−4 hours.

3. Add 100 μL STOP solution to each well and incubate for 1 hour at room temperature.

4. After 1 hour, mix the contents so that a uniform colored solution is obtained. The multichannel pipette can be used to do this, but be careful not to cause bubbles at the surface to avoid interfering the absorbance measurement.

5. Measure the absorbance using a plate reader at a wavelength of 560 nm and at a reference wavelength of 750 nm.

**Supplementary figures**


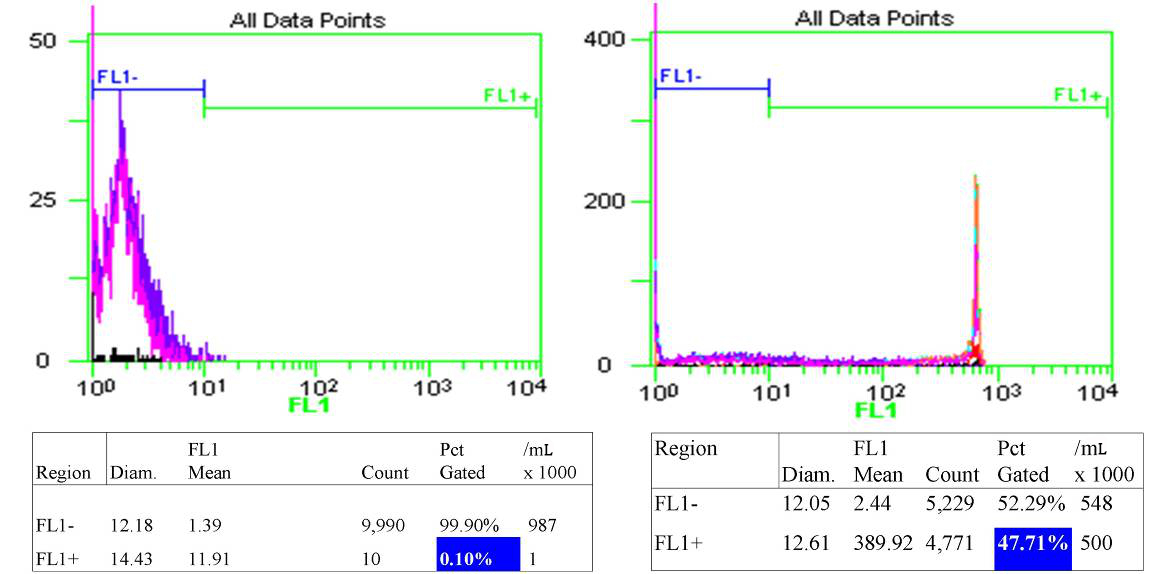


**Figure S1.** **Examples of determination of the transfection efficiency using FACS.** (Left) Graph obtained by FACS measurement of transfection efficiency at N/P ratio of 0. (Right) Graph obtained by FACS measurement at N/P ratio of 160 for PEI_60kD_. The lower significance level of the transfection efficiency is determined according to the left graph, which is applied in the measurements of the transfection efficiencies at N/P ratio of 160 for PEI_60kD_ in the right graph. The tables below the graphs show the transfection efficiencies of 0.10% (left) and 47.71% (right) in blue-colour numbers.


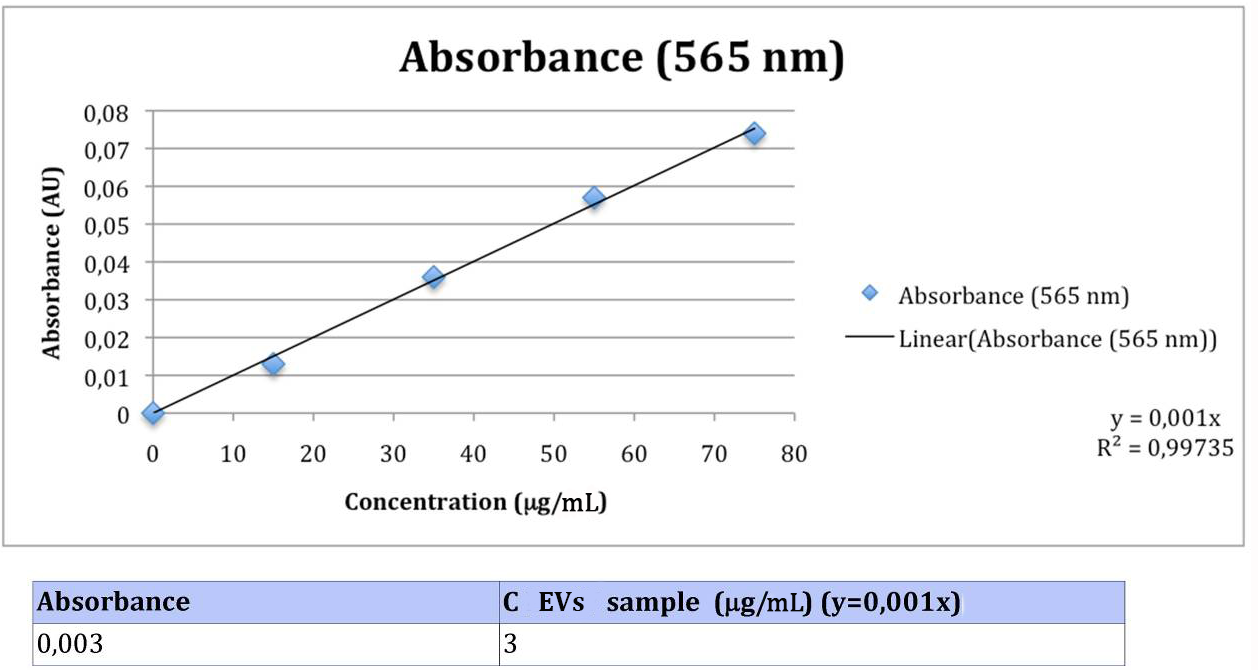


**Figure S2.** The results of protein content in EVs solution detected using the Bradford assay.


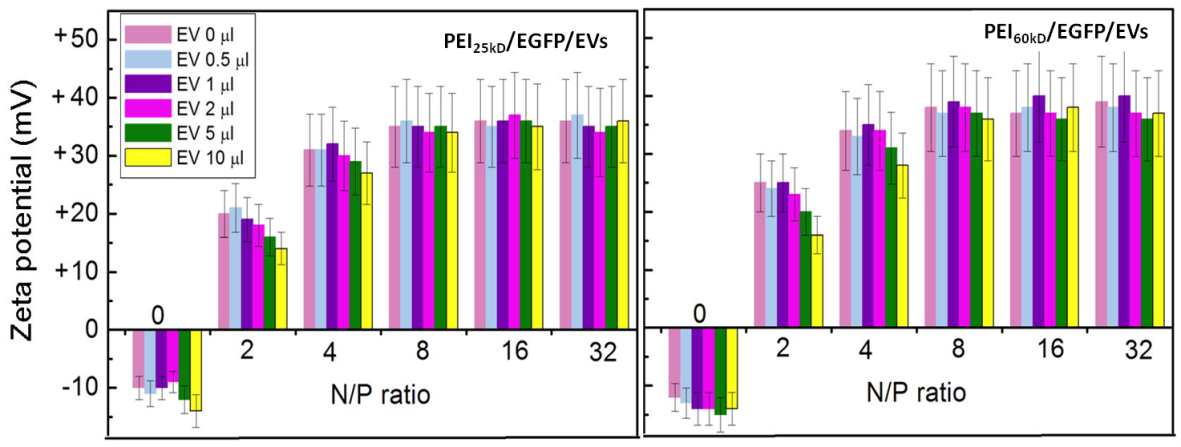


**Figure S3.** The zeta potential of PEI_25kD_/EGFP/EVs and PEI_60kD_/EGFP/EVs at different N/P ratio and EV volumn.

**
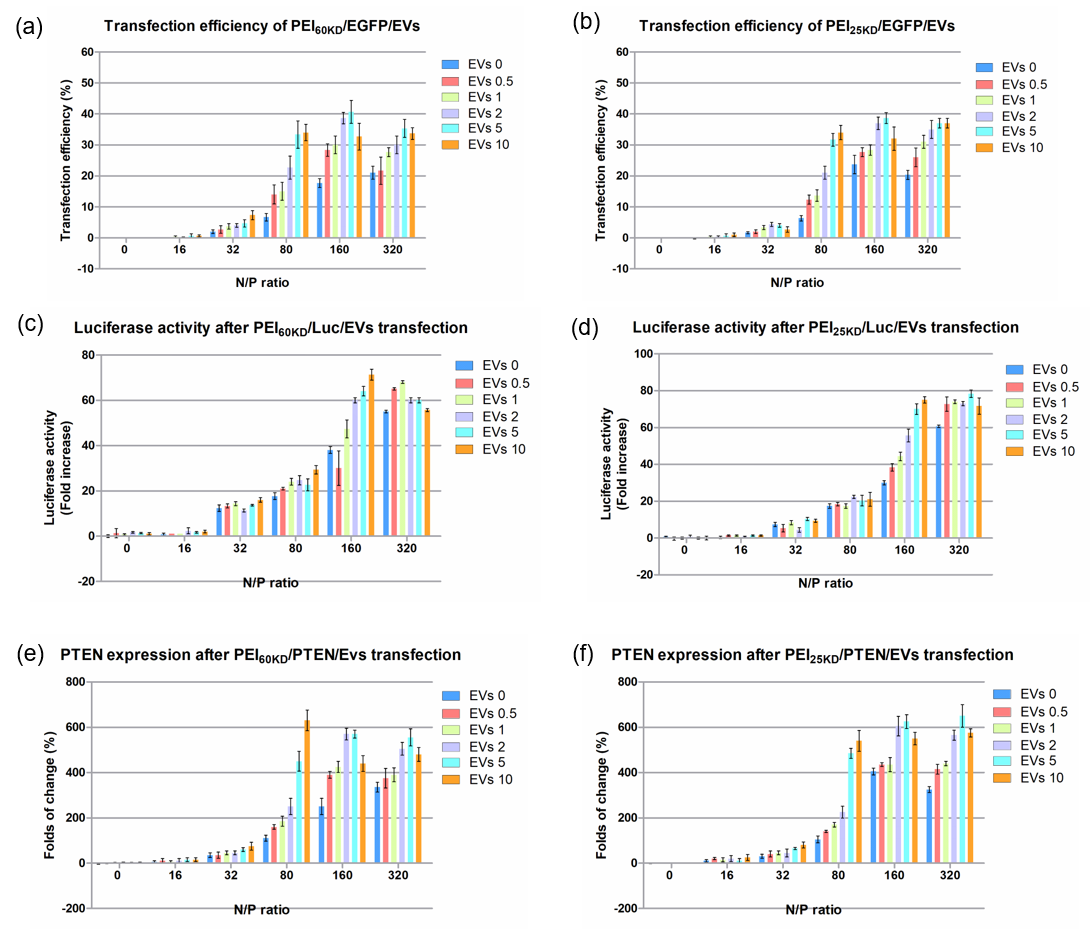
**

**Figure S4.** Transfection efficiency of A549 cells transfected with (**a**) PEI_60kD_/EGFP/EVs and (**b**) PEI_25kD_/EGFP/EVs, determined by FACS assay; Luciferase activity of A549 cells transfected with (**c**) PEI_60kD_/Luc/EVs and (**d**) PEI_25kD_/Luc/EVs, determined by luciferase assay; PTEN expression of A549 cells transfected with (**e**) PEI_60kD_/PTEN/EVs and (**f**) PEI_25kD_/PTEN/EVs, determined by PCR assay; at a post transfection time of 48 hours for different N/P ratios. EV solution of different volume (0, 0.5, 1, 2, 5, and 10 μL) with protein concentration of 3 ng μL^-1^ were added into each well of the 96-well plate.


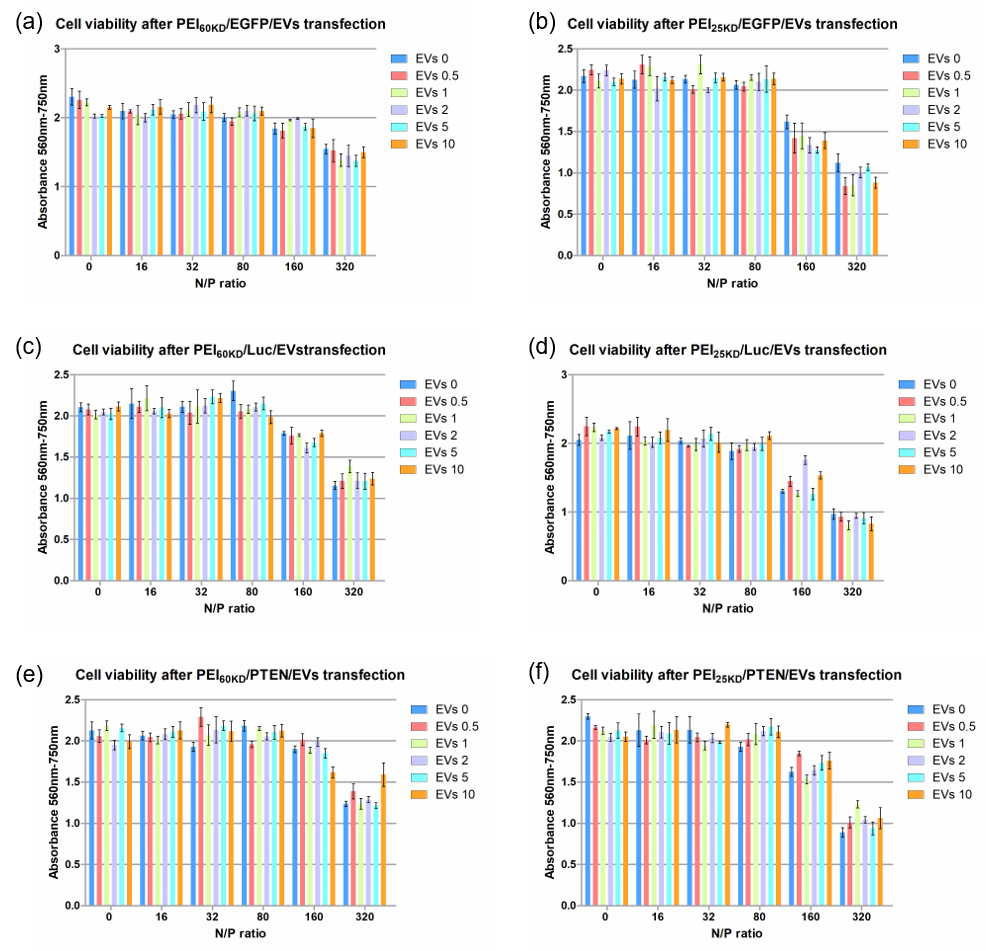


**Figure S5.** Cell viability of A549 cells transfected with (**a**) PEI_60kD_/EGFP/EVs, (**b**) PEI_25kD_/EGFP/EVs, (**c**) PEI_60kD_/Luc/EVs, (**d**) PEI_25kD_/Luc/EVs, (**e**) PEI_60kD_/PTEN/EVs and (**f**) PEI_25kD_/PTEN/EVs, determined by MTT assay, at a post transfection time of 48 hours for different N/P ratios. EVs solution of different volume (0, 0.5, 1, 2, 5, and 10 μL) was added into each well of the 96 well plate. The protein concentration in EV solution was 3 ng μL^-1^.
